# Encouraging research on recursive thinking through the lens of a model of the spread of contagious diseases

**DOI:** 10.1007/s11858-022-01354-6

**Published:** 2022-05-03

**Authors:** James Sandefur, Alfred B. Manaster

**Affiliations:** 1 Georgetown University, Washington, DC USA; 2 University of California San Diego, La Jolla, CA USA

**Keywords:** Discrete mathematics, Recursive thinking, Mathematical induction, Difference equations, Epidemics

## Abstract

Recursive reasoning is a powerful tool used extensively in problem solving. For us, recursive reasoning includes iteration, sequences, difference equations, discrete dynamical systems, pattern identification, and mathematical induction; all of these can represent how things change, but in discrete jumps. Given the school mathematics curriculum’s later emphasis on calculus—the mathematics of change in continuous contexts—it is surprising that the curriculum seems to neglect recursive thinking after the early grades. Research shows that recursion supports the learning of algebra among younger students, but the lack of similar research with older students is concerning. In this paper we suggest possible affordances from teaching recursive modeling, including a basic model of the spread of contagious diseases. We also discuss different ways to present these models at various points in the curriculum that might develop connections between mathematics and the real world, and support students’ learning of mathematics. This leads to what we, as mathematicians, think would be interesting research questions for mathematical educators.

## Introduction

This article’s main purpose is to promote research on the effects of students learning recursive thinking and understanding recursion. By recursion, we mean, roughly speaking, systematic sequential change, as opposed to the continuous change studied in calculus. Recursion includes iteration, sequences, difference equations, discrete dynamical systems, and mathematical induction.

Our discussion focusses on a basic model of the spread of contagious diseases—presented in some detail and in ways that might be suitable in various places in a mathematics curriculum. This model is one of many recursive mathematical models that students could study. Along the way, we suggest some possible affordances in student learning and positive effects on students’ attitudes toward mathematics. We also briefly discuss other recursive models that could engage students, show connections between mathematics and the real world, and support students’ learning of mathematics through the use of spreadsheets. This discussion raises important questions we hope mathematics education research will investigate.

We promote the study of recursive thinking because we believe the incorporation of iteration, recursion, and difference equations into school mathematics can lead to, among other things,

- easier and deeper learning of many topics in the current mathematics curriculum,
- increased student interest in and engagement with mathematics,
- expanded understanding of the nature of mathematics,
- better appreciation of the interplay between mathematics and other disciplines, and
- understanding of how mathematics can support many career choices, leading to a desire to continue learning mathematics.

Unfortunately, these beliefs are only marginally supported by the limited research on the relationship between mathematical learning and the development of recursive thinking. In this paper we will indirectly reference these potential broad benefits of learning recursive thinking as we analyze a particular yet generalizable recursive model and suggest many specific researchable affordances that we hope will be pursued.

## Background and literature review

We will not repeat the general argument for an increase in the teaching of discrete mathematics in the curriculum, but instead focus on one particular discrete topic, recursive thinking. By recursive thinking, we mean thinking in terms of cause-and-effect in which the same process keeps being repeated. For example, if a student continued to fold a piece of paper in half, the number of layers would double after each fold. A well-known recursive equation generates the Fibonacci sequence in which each number is the sum of the previous two.

As another example, to compute *n!*, we could start with *a*_*1*_ = *1*, then compute *a*_*2*_ = *2a*_*1*_, then *a*_*3*_ = *3a*_*2*_, and so on. Computer scientists call this iteration. They define recursion as a top-down approach. For example, to compute *n!*, they start with *n*, then go backwards to *n(n-1)!*, and continue until they get to $1$. To avoid confusion, we will call this top-down recursion or TD recursion, for short.

Top-down recursive programming is quite important in learning computer science and has been studied extensively. Papert (1980) promoted learning computer literacy in primary and secondary education, using the TD recursive software Logo. Ansai and Uesato (1982) showed that adolescents understood TD recursion much better if they were first familiar with iteration. Kurland and Pea’s (1985) study of 8–12-year-old students who had a year of experience working with Logo indicates that most avoid all but simple iterative programs. Many computer scientists have suggested a variety of models for teaching TD recursion, such as the Tower of Hanoi or the Eight Queens problem. One interesting model that takes advantage of the top-down nature of the problem is parking cars (Wirth, 2008). Computer scientists tend to agree that teaching TD recursion is quite difficult. McCauley et al. (2015) gives a nice survey of the literature on the difficulty of teaching TD recursion. They note that hundreds of articles have been published related to the teaching and learning of TD recursion, but there are less than 50 research results.

This article focuses on iteration and not top-down recursion, using the terms “recursion” and “iteration” interchangeably.

There is some evidence that iterative tasks support elementary and middle school students’ transition from computation to algebra. Blanton and Kaput (2005) indicate that a combination of iterative contextual problems combined with covariational reasoning can help students develop algebraic thinking, even as early as the second grade. In the January 2008 issue of ZDM—Mathematics Education, several articles (Amit & Neria, 2008; Carraher et al., 2008; Radford, 2008; Rivera & Rossi-Becker, 2008; Steele, 2008; Yeap & Kaur, 2008) give evidence that iterative problems support learning algebra by students in grades 3 through 8. The contextual and often hands-on iterative problems discussed in these papers supported the learning of algebra as students went from the iterative approach to developing formulas and generalization.

Given this evidence that iterative tasks support learning algebra and that the curriculum includes a focus on preparing for the study of continuous change—calculus and derivatives—the small number of studies on the continued use of iterative tasks at the secondary level and beyond is surprising. In one study (Weigand, 2004) grade 12 students and university students used spreadsheets to study sequences, such as linear growth, *a*_*n*+*1*_ = *a*_*n*_ + *B*, exponential growth *a*_*n*+*1*_ = *Aa*_*n*_, and logistic growth *a*_*n*+*1*_ = *a*_*n*_ + *P(B-a*_*n*_*)*; they also explored finite differences, i.e., ${a}_{n+1}-{a}_{n}$, for different sequences. Weigand noted that success required a well-structured learning process and care to ensure that students not work on only a symbolic level without understanding. A second study (Harel, 2001) considers the introduction of induction through the use of naturally recursive contextual scenarios.

Given the studies showing iterative problems support learning algebra and the importance of top-down recursion for computer science, we wonder why almost the only iterations seen in the secondary curriculum are Fibonacci sequences and Pascal’s triangle. We think students’ schooling should build on the study of iterative change in the early grades.

We agree with the conclusions of Lannin et al. (2006) who studied the relationship between students developing explicit and recursive rules for a variety of contextual situations. In particular, they concluded that “As such, students should be able to reason using explicit and recursive rules, and recognize the connections that exist between these two types of rules.” While their study was with pre-algebra students, we believe the conclusions apply to secondary and college students.

The iterative problems used to promote algebraic thinking among pre-algebra students are usually engaging and playful for the students. Often, they are hands-on, with objects that are built with toothpicks or patterns made from stickers (Radford, 2008; Yeap & Kaur, 2008). We propose going beyond these simple iterative problems by introducing important iterative real-world models to secondary students to promote a more advanced and deeper algebraic understanding. We raise questions about this proposal that we hope the mathematics education community will address.

## A simple contagious disease model

In an effort to show the possible affordances arising from recursive models, we present a basic model of the spread of a contagious disease and consider various presentations and perspectives of it in following sections. This model, appropriate for both middle and secondary school students, is one representative of the type of recursive mathematical model that could be introduced in school mathematics. It is a simplified version of the susceptible/infected/recovered (SIR) models (Eggo, 2018; Smith & Moore, 2001), variations of which are currently in common use in epidemiology. Many of these models are continuous differential equations models. In practice, the implementation of these models is usually discrete in the sense that a computer applies discrete iterations to go forward in time under a variety of assumptions, much as our model does. In fact, the spread of a disease has both continuous and discrete aspects, neither being perfect. This is another reason students should study discrete mathematics as well as continuous mathematics.

This model will demonstrate that modeling itself is an iterative process. While a great deal can be learned from a simple model, the repeated replacement of simplifying assumptions with more realistic assumptions can lead to more realistic models, providing additional insight along the way.

For our model, we consider discrete intervals of time: our particular interval of time is the length of time a cohort of recently infected individuals is contagious. The model develops a projection of the spread of a contagious disease under the following assumptions that are easy for students to understand.

- There is a fixed population size.
- In each time interval, the population consists of susceptible people, contagious people, and immune people.
- Each contagious person is contagious for just one time-period, then becomes immune and remains immune.
- Each contagious person comes into contact with the same number of people while being contagious.
- Each susceptible person who comes into contact with a contagious person has the same probability of becoming infected.
- Each susceptible person who becomes infected is contagious in the next time-period.

As the model is developed, students can discuss how realistic they believe these assumptions are, and might suggest other assumptions or variations on these assumptions. For example, this model avoids mortality.

To get a sense of the model, students could construct three sequences related to the spread of a contagious disease. The first sequence is the number of susceptible individuals initially (i.e., the size of the first cohort of susceptible persons), just after the first time-period, just after the second time-period, and so forth. The second and third sequences are the numbers of contagious and immune people (i.e., the sizes of the cohorts of contagious and immune people) respectively during each time-period. Unlike many previously considered sequences, these are not arithmetic or geometric: they cannot be constructed by looking at the pattern of numbers but must be constructed by considering the contextual situation—unusual for school mathematics, but common in applied mathematics.

**Fig. 1:**
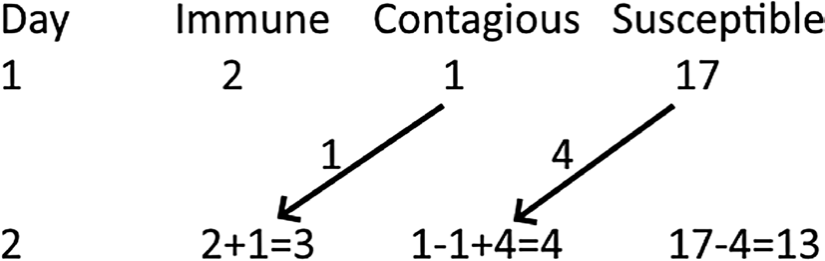
Example of simulation of contagious disease. Arrows indicate students moving from one cohort to another

The method of presentation should depend on the students’ ages, backgrounds, and abilities. For students who have not studied algebra, it is possible to start with a classroom simulation in which the time-period for contagion is 1 day. For example, on the first day, 1 student is contagious, 2 students are immune, and 17 are susceptible. They could also assume that each day, each contagious student contacts 5 other students and each susceptible student has a 50% chance of becoming infected if in contact with at least one contagious student. They could simulate this by drawing 5 names for each contagious person and flipping a coin for each susceptible person to determine if they became infected. Note that in this simulation, a susceptible person could come into contact with more than one contagious person. One possible result is seen in Fig. 1, where we suppose that after the simulation, 4 students become contagious on Day 2.

Students in early grades could make a table of their results. Students in later grades could use sequence notation or function notation, such as ﻿

$$s_{1}= 17, \, c_{1}=1, \, i_{1} = 2, \, \text{or}\,  s{(1)} = 17, \, c{(1)} = 1, \, i{(1)} = 2.$$

Once students understand the context of the contagious disease model, they can start to develop a larger example using our assumptions. For example, consider a population of 5000, of which initially none are immune, 100 are contagious, and 4900 are susceptible to the disease. These are the first cohorts. To develop the model, the students need to know the contact number, that is, how many people each contagious person contacts. They also need to know the chance of infection, that is, the probability that a susceptible person becomes infected after contact with at least one contagious person. While it is not realistic, for ease of computation we add an additional assumption:

- Each susceptible person comes into contact with at most one contagious person.

With a large population and a relatively small number of infected, the results would be similar. For this example, suppose that, on average, each infected person contacts eight people, and the chance of infection is 20%. Students should be able to compute that the 100 contagious people come into contact with $8\times 100=800$ people. Of these, students compute that the number of susceptible people contacted is

$$800\left( {\frac{4900}{{5000}}} \right) = 784.$$

Finally, the number of these susceptible people who become infected, and therefore contagious is

$$0.2 \times 784 = 156.8,$$

which they might round to 157. This leaves 4900–157 = 4743 susceptible people. The students now have computed the first and second cohorts, as seen in Table 1.

**Table 1:** First several cohorts of susceptible, contagious and immune people with population 5000, contact number 8, and likelihood of infection 20%

Time Period	1	2	3	4	5
Contagious	100	157	238	343	457
Susceptible	4900	4743	4505	4162	3705
Immune	0	100	257	495	838

The development of Table 1 uses proportions in computing the number of susceptible people contacted by contagious people, and averages or simple probability to compute the expected number of contacted susceptible people who become contagious. The class could discuss how realistic these computations are. They might complain that we are assuming each contagious person comes into contact with exactly 8 different people and there is no overlap in those contacted by different infected individuals, which is not realistic. On the other hand, if the population is large enough, does this use of average impact the result significantly? Students should also consider that the 20% chance of infection is an approximation. This means that for cohort 2, we expect, on average, 157 contagious people, but are aware it could be slightly higher or lower.

Students should now use the same process to compute the third, fourth and fifth cohorts, seen in Table 1. This simple model provides an opportunity to talk about averages, with the computed numbers being an expected result on average, not necessarily an exact result.

Other models than contagious diseases can be developed with students, such as a variety of models of population growth (Weigand, 2004). A population growth model with fixed growth rate is *a*_*n*+*1*_ = *Aa*_*n*_, which would result in exponential growth. The logistic equation *a*_*n*+*1*_ = *a*_*n*_ + *P(B-a*_*n*_*),* has a linearly decreasing growth rate, and generally has no algebraic formula for its solution. We think it is important that students realize not all problems can be represented by explicit algebraic solutions and some must be studied using other methods, such as recursion and/or spreadsheets.

## Recursion and the use of spreadsheets

The ease with which computations can be done with spreadsheets in studying iterative problems supports students learning algebra (Tabach et al., 2013). While not being research studies, Cornell and Siegried (1991) and Maxim and Verhey (1991) have also argued that the learning of traditional math and algebra is supported through teaching recursion combined with the use of spreadsheets. Their arguments support our view that the use of spreadsheets combined with recursive thinking is potentially a powerful educational tool in developing reasoning skills and meaningful applications while supporting the learning of traditional mathematics. These papers describe how the use of spreadsheets can be used in a variety of recursive models.

This section describes how spreadsheets might be used similarly for our contagious disease model. Students might realize that the same computations are repeated for each cohort. In particular:

- the estimated number of individuals contacted by the current cohort of contagious people is the number contagious multiplied by the contact number;
- the estimated number of susceptible people coming into contact with a contagious person in the current cohort is found using proportions;
- the estimated number of contagious people in the next cohort is the product of the number of contacted susceptible people and the chance of infection;
- the estimated size of the next cohort of susceptible people is the result of subtracting the number of newly contagious people from the previous cohort of susceptible people; and
- the estimated size of the next cohort of immune people is the sum of the previous cohort of contagious people and the previous cohort of immune people.

Here is where students can discover the power of arithmetic by using spreadsheets. Instead of continuing to compute by hand, the students can make a simple spreadsheet like Table 2. This spreadsheet is just a straightforward translation of the steps listed in constructing Table 1.

**Table 2:** Spreadsheet template for contagious disease model

	A	B	C	D
1	Cohort	Contagious	Susceptible	Immune
2	1	100	4900	0
3	= A2 + 1	= 0.2*8*B2*(C2/5000)	= C2-B3	= D2 + B2

By copying down the last row, the spreadsheet will repeat all the computations, although some values will not be whole numbers. From looking at the numbers in the spreadsheet, students can see that the number of contagious people increases to a maximum of 548 in cohort 7, then decreases to 0. Similarly, the total number of immune people levels off at 3540. This means that a total of 3540 people eventually had the disease and became immune, while 1460 escaped being infected. Students could discuss if it makes sense to look at the limit of the total immune in the spreadsheet, or if they should stop computing once the number of infected is less than 0.5, which occurs in the 19^th^ time period with a total of 3539 being immune, after rounding.

How can teachers determine whether using whole number quotients or rational values is more effective for their students? Some students may find whole numbers more meaningful and can use them to compute the values of the first few cohorts. Using rational numbers can reinforce that the results are only approximations. Either way, it is important for students to understand that rounding will not have much impact for relatively large populations, that the numbers are averages, and that variation is expected.

We believe that the use of spreadsheets will help transition the curriculum from the current formula-based system toward a more process-based system. Time, as well as careful observation by researchers, is needed for us to learn how realistic our belief is.

## Algebraic notation for recursion and difference equations

As described in §2, there is evidence that students as early as the second grade can discover recursive patterns in contextual situations and use the recursive pattern to develop algebraic formulas. Of particular interest is how, in several studies, students developed linear functions from recursive patterns that involved repeated addition. In one study (Carraher et al., 2008), third grade students considered the number of people who could sit around a long collection of tables. Each time a table was added, two more people could sit. Students translated `add 2’ into $2t+2$, thus translating repeated addition into multiplication, developing an algebraic formula. Rivera and Becker (2008) conducted a 3-year longitudinal study of middle school students in which they developed linear functions using different methods. Some students used a constructive method in which the same amount was added each time, resulting in the formula $3n+1$ for counting the smallest number of toothpicks needed to make *n* connected squares. Other students used a deconstructive method in which they had overlapping parts in which they had to then subtract an appropriate amount, yielding $4n-(n-1)$ for the same problem. Amit and Neria (2008) had a similar result with sixth and seventh grade students who in studying a tiling problem observed that after each iteration, the number of tiles increased by 8 leading to correct formulas, such as $8n+8$ or $16+[\left(n-1\right)8]$, depending on how they saw the problem. In that study and a study by Yeap and Kaur (2008) with fifth grade students, nonlinear problems were considered, in which the amount added each time increased, resulting in a quadratic expression. This shows the continued development of the relationship between recursion and algebra as students advance.

These studies and others indicate that students look for patterns, which they should continue to do. Several people have proposed different methods for connecting recursion and algebra. In the Now/Next method (Hart, 1997; Hart & Martin, 2016; Hart et al., 2008; Lannin et al., 2006; Martin & Hart, 2012), students would write the arithmetic sequence for the toothpick problem in Rivera and Becker (2008) as

1 $${\text{Next}} = {\text{Now}} + {3}$$

while the use of difference equations and sequence notation (Dossey, 1991, 1997) would result in

$$s_{n + 1} = s_{n} + 3,$$

where $s_{o} = 1$ for sequence 1). In this case, the closed-form solution

$$s_{n} = 3n + 1$$

is easily found, as did the students in that study, demonstrating that repeated addition can lead to multiplication.

Similarly, repeated multiplication leads to exponentiation, as seen by either using the Now/Next method,

2 $${\text{Next}} = {\text{3Now}},$$

or the first order difference equation

$$s_{n + 1} = 3s_{n} .$$

Letting $s_{o} = 1$ results in the closed-form exponential solution

$$s_{n} = 3^{n} .$$

Such exponential results arise in the mathematics of finance and in population dynamics.

These sequences have the additional advantage that they can be developed within interesting contextual settings. For example, the buildup in the plasma of a repeatedly taken medicine (Sandefur et al., 2018) in which *r* is the fraction eliminated each time-period and *d* is the repeated dose results in the recursive equation

$${\text{Next}} = \left( {1 - r} \right) \times {\text{Now}} + d.$$

This medicine model can also be written as a difference equation using sequence notation,

3 $$s_{n + 1} = \left( {1 - r} \right)s_{n} + d .$$

As students progress in their mathematics and the recursive problems become more complex, the sequence notation style must replace the Now/Next method. A difference equation of the form

4 $$s_{n + 1} = s_{n} + {\text{f}}\left( n \right),$$

where the amount being added is a function of $n$, is called nonhomogeneous. In Yeap and Kaur (2008) students developed both a recursive and quadratic closed form solution when adding consecutive odd integers, ${s}_{n+1}={s}_{n}+2n+1$. In Amit and Neria (2008), students generated both the recursive and quadratic formula for the total number of candles lit over Hanukah where each day one more candle is lit than the day before, ${s}_{n+1}={s}_{n}+n+1$. Some additional examples of difference equations similar to﻿ Eq. 4 are

- counting how many handshakes occur among *n* people if each pair shakes hands exactly once (Sandefur, 1997),
- counting the number of seats in a theater (Burrell et al., 1991), and
- a model of skydiving and acceleration of gravity (Hart et al., 2008).

The sequence approach works well for second order models, such as the Fibonacci sequence, written as

$$s_{n + 2} = s_{n + 1} + s_{n}$$.

Our contagious disease model involves several changing variables with each value depending possibly on previous other values. It is described by the system of equations

$$\begin{array}{*{20}c} {c_{n + 1} = \left( {0.2} \right)\left( 8 \right)\left( {c_{n} } \right)\left( {\frac{{s_{n} }}{5000}} \right)} \\ {s_{n + 1} = s_{n} - c_{n + 1} } \\ {i_{n + 1} = i_{n} + c_{n} } \\ \end{array}$$

or, after substituting,

5 $$\begin{array}{*{20}c} {c_{n + 1} = \left( {0.2} \right)\left( 8 \right)\left( {c_{n} } \right)\left( {\frac{{s_{n} }}{5000}} \right)} \\ {s_{n + 1} = s_{n} - \left( {0.2} \right)\left( 8 \right)\left( {c_{n} } \right)\left( {\frac{{s_{n} }}{5000}} \right)} \\ {i_{n + 1} = i_{n} + c_{n} } \\ \end{array}$$

which is the more traditional form.

## Graphing

We believe discrete models combined with the use of spreadsheets can help students better understand graphs of functions because they can generate the graphs themselves, within a contextual setting. In these cases, the variables have meaning for the students.

Some spreadsheet models result in traditionally studied graphs. Parabolic graphs result from difference equations of the form Eq. 4 where $f(n)$ is linear. Exponential graphs occur for models of radioactive decay and for models of the buildup and elimination of drugs (Sandefur, 1992; Sandefur et al., 2018).

**Fig. 2:**
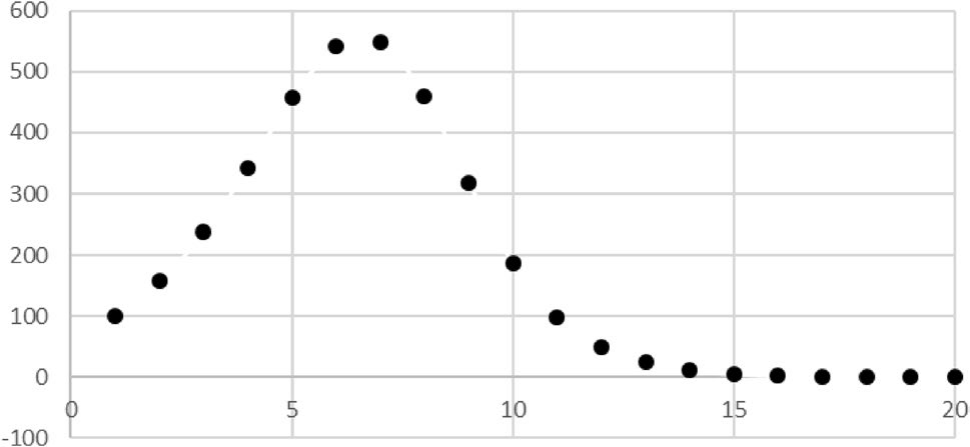
Number of infected in each cohort with population 5000, contact number 8 and a likelihood of infection of 20%

**Fig. 3:**
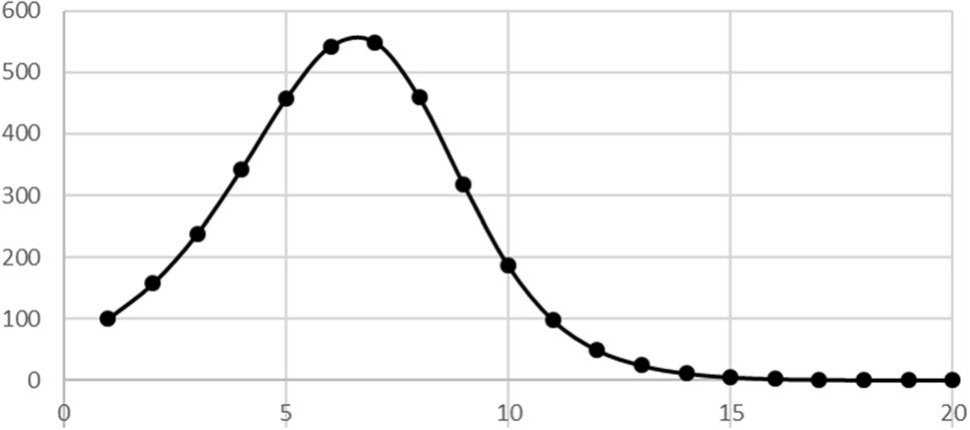
Number infected in each cohort with points connected by curves

Beyond that, some recursive models result in non-traditional graphs; it seems to us that this can enrich students’ understanding of and appreciation for mathematics. The analysis of the spread of contagious diseases leads to nontraditional graphs. Using our model of contagious disease spread Eq. 5, we could graph the number of contagious people in each time-period, as seen in Fig. 2. We only plotted points because we are only generating the number of contagious persons in discrete time-periods. We could also smoothly connect the points, as in Fig. 3. This suggests that while we are considering discrete time intervals, there is probably a more gradual spread of infection occurring within each time-period so that the number of contagious people is increasing or decreasing during each time-period. Through models such as this, students can generate and discuss graphs that are not graphs of the standard functions (polynomials, rational functions, exponential functions) normally graphed in school. By considering graphs such as Fig. 3, students can interpret the meaning of the variables from the graphs. In this way, graphs represent more than just a relationship between two abstract variables. The fact that the students generate the graphs should give them more ownership over and investment in the graphs than they have when interpreting a graph given in a textbook.

An important aspect of graphing that arises from recursive models is the study of asymptotes. Our experience indicates that students, even college students, often do not understand the significance of an asymptote. The exponential closed form solution to the medicine example 3) is difficult for students to discover. If students make graphs of solutions using spreadsheets, they will see that over time, the amount of drug will level off at a horizontal asymptote. This asymptote is the desired equilibrium amount of medicine in the plasma for that given dose. This is easy for students to understand. For Eq. 3, this equilibrium is achieved when ${s}_{n+1}={s}_{n}$ (or Next = Now), so to determine this, students substitute *x* for ${s}_{n+1}$ and ${s}_{n}$ and solve

$$x = \left( {1 - r} \right)x + d$$

for *x*, giving $x=d/r$. In reality, *x* and *r* are usually known and this formula is used to find the appropriate dose, *d* = *xr*. Note that the important information has been found without the use of a closed formula.

Another example of meaningful asymptotes arises from population growth models, such as the logistic equation,

6 $$p_{n + 1} - p_{n} = r\left( {1 - p_{n} /L} \right)p_{n}.$$

The variable *r* is called the intrinsic growth rate and *L* is called the carrying capacity. If the intrinsic growth rate *r* is reasonably small, graphs of $p_{n}$ have a horizontal asymptote at $p = L$. Students could discover that this is reasonable since this is the value for which the change in population, $p_{n + 1} - p_{n}$, is zero. The horizontal asymptote is found by solving the equation

$$x - x = r\left( {1 - x/L} \right)x,$$

where *x* is substituted for ${p}_{n}$ and ${p}_{n+1}$ in Eq. 6. Note that there is a second solution, $p=0$, which is also an equilibrium value: if the population is extinct, it remains extinct. This shows the difference between a stable equilibrium, *L*, and an unstable equilibrium, 0. Thus, solving equations relates to asymptotes and gives a reason for understanding both.

A variation on the medicine problem (Sandefur et al., 2018) assumes that some of the medicine is being absorbed from the plasma into the liver and vice versa, which is true for vitamin A. This gives a pair of equations, such as

7 $$\begin{array}{*{20}c} {d_{n + 1} - d_{n} = } & {a - bd_{n} - rd_{n} + sl_{n} } \\ {l_{n + 1} - l_{n} = } & { - sl_{n} + rd_{n} } \\ \end{array}$$

where ${d}_{n}$ and ${l}_{n}$ are the amount of the drug in the plasma and liver, respectively, after *n* time periods. For these equations, *a* is the size of the dose of medicine, *b* is the fraction eliminated by the kidneys, *r* is the fraction absorbed from the plasma into the liver and *s* is the fraction absorbed from the liver into the plasma. A spreadsheet graph will show horizontal asymptotes for both ${d}_{n}$ and ${l}_{n}$. By substituting *x* for ${d}_{n}$ and ${d}_{n+1}$, and *y* for ${l}_{n}$ and ${l}_{n+1}$ and solving the pair of linear equations, we hope students will come to understand equilibrium: these values are the equilibrium amounts of the medicine in both the plasma and liver. We also intend that students understand how these values are related.

**Fig. 4:**
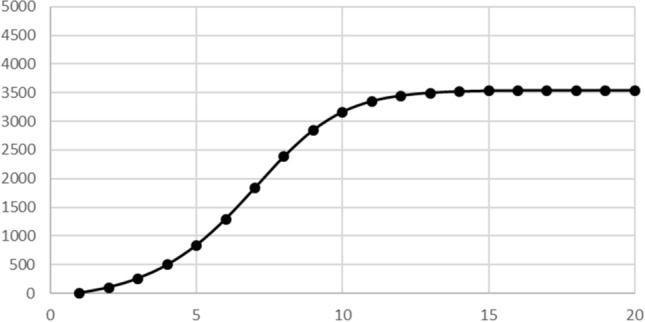
Immune people over time with population 5000, contact number 8 and a chance of infection of 20%

Figure 4 is the graph of the sizes of the immune cohorts in our contagious disease model, which are also the total numbers infected over time. It has the traditional S shape with a horizontal asymptote, often associated with contagious disease models and population growth models. The asymptote in this case is different from the medicine models and the population model in that there is no unique solution for the equilibrium value. In particular, to solve for the equilibrium values for the contagious disease model 5), substitute *x* for ${s}_{n}$ and${s}_{n+1}$, *y* for ${c}_{n}$ and ${c}_{n+1}$, and *z* for ${i}_{n}$ and${i}_{n+1}$, to get the equations

$$\begin{array}{*{20}c} {y = \left( {0.2} \right)\left( 8 \right)\left( y \right)\left( \frac{x}{5000} \right)} \\ {x = x - \left( {0.2} \right)\left( 8 \right)\left( y \right)\left( \frac{x}{5000} \right)} \\ {z = z + y} \\ \end{array}$$

The third equation implies $y=0$, which makes sense: for the epidemic to die out, there can be no contagious people. This means that the values for *z* and *x* can be anything. Here is a natural example of a system of three equations and three unknowns which, mathematically, has an infinite set of solutions. On the other hand, since the equilibrium for ${s}_{n}$ and ${i}_{n}$ must be integers and ${s}_{n}+{i}_{n}$ must equal the size of the population, realistically speaking, there is not really an infinite set of equilibrium values.

Figure 4, like the medicine and population models, is an example in which the horizontal asymptote has real-world significance. Unlike the medicine models and the population model, the asymptote for the contagious disease model Eq. 5 cannot be determined from the equations alone, but also depends on sizes of the initial cohorts.

## Understanding parameters: physical distancing

The Common Core State Standards for Mathematics (http://www.corestandards.org/Math/) recommends that students understand the value of parameters in a variety of contextual situations. For example, while students may determine that a parameter shifts a parabola vertically or horizontally, they should see the value of parameters in a broader, real-world context. Watson and Chick (2011) discuss an exploration of polynomials involving a variety of parameters, but the goal of the example was to construct a polynomial with certain characteristics, not to see how the parameters affected a realistic outcome. On the other hand, Lingefjӓrd (2006) discusses models with a variety of parameters related to real outcomes, but the models are at an advanced level, inappropriate for school mathematics. Berry (2002) argues that students can explore simpler models if they use a Computer Algebra System, and gives an example of the design of a drink can.

Most of the recursive models described previously in this article have one thing in common, one or more parameters that affect the outcome of the model. For example, drug model Eq. 3 has two parameters, the elimination rate $r$, over which we have little control, and the size of the dose of medicine, $d$, over which we do have control. Changing these parameters within a spreadsheet changes both the horizontal asymptote and the rate at which we approach this asymptote. Thus, recursive modeling combined with the use of spreadsheets gives a simple way for students to explore mathematically where their explorations lead to insight into a model of interest.

Our infectious disease model provides many opportunities for students to explore the effects of parameters. For example, in Table 2 and Fig. 4, we see that, with a population of 5000, a contact number of 8 and a likelihood of infection of 20%, the total eventually infected stabilizes at about 3540. Since $${\raise0.7ex\hbox{${3540}$} \!\mathord{\left/ {\vphantom {{3540} {5000}}}\right.\kern-\nulldelimiterspace} \!\lower0.7ex\hbox{${5000}$}} = 0.708$$, about 70% of the population becomes infected. This suggests that the population will develop herd immunity when 70% of it is immune.

One advantage of using spreadsheet models combined with recursive thinking and traditional mathematics is that surprising results can often occur. That is the case for the study of contagious diseases: simple explorations with parameters lead to some interesting results. For example, students could try different values for the contact number, say 7 instead of 8. This is easy to accomplish using a spreadsheet. The result is that only 2865 eventually become infected, telling us that $${\raise0.7ex\hbox{${2865}$} \!\mathord{\left/ {\vphantom {{2865} {5000}}}\right.\kern-\nulldelimiterspace} \!\lower0.7ex\hbox{${5000}$}} = 0.573$$, or just over 57% of the population, become infected. This is in contrast to 70% with contact number 8. Also, the peak number infected in any time-period is smaller, 339 versus 548 in cohort 7. This demonstrates to students how the effect of physical distancing works. Again, students could try larger and smaller numbers for the contact number to see the effects. Students could discuss how we could reduce the contact number; one example might be limiting the size of gatherings. Different surprising results occur for the ﻿logistic equation Eq. 6 in which case, erratic (chaotic) behavior occurs for growth rates that are too large.

Instead of decreasing the contact number, students could consider the impact of changes in the likelihood of infection by using different values in the spreadsheet model. They could discuss ways to decrease the likelihood of infection such as by people being careful wearing masks or staying a little further apart when they do come into social contact with each other, just as they could discuss the medicine model with different size doses and different elimination rates.

Class discussions could result in students seeing that the product of the contact number times the likelihood of infection plays a key role in the examples; for instance, a contact number of 16 and likelihood of infection of 10% results in the same total number eventually immune, 3540. If students consider the spreadsheet model, they should see why this is. In cell B3 of Table 2, what is important is the number

$$1.6 = 8 \times 0.2 = 16 \times 0.1.$$

We will call the product of the contact number and the likelihood of infection, the **reproductive number**. Note that this is only the average number of contagious people in one of our time-periods for each contagious person in the previous time-period if the immune cohort of the previous time-period is empty. In our computations, we might use this one number instead of using both the contact number and the likelihood of infection, but using these two parameters gives us more insight into the model. This is similar to using a growth rate instead of a birth rate and death rate in population models.

We do note one caveat in these explorations. If the reproductive number is too large, 1.8 in our example, then the number of people contacted by contagious people in some cohorts might exceed the number of people in the population, 6093 in cohort 5 for this example. A refinement of the model can limit the number of contacts to the size of the population.

Once students understand the effect of the reproductive number on the total eventually infected, they can continue to take advantage of the use of spreadsheets to reexamine the model by varying other parameters in the model such as population size, initial number infected, and likelihood of infection. For example, if they use a different initial contagious cohort, say 50, the model predicts that only 3487 people catch the disease, which is not much different than the 3540 who catch the disease with an initial cohort of 100. In fact, if only 1 person is initially infected, 3432 people still become infected. Thus, the initial cohort of infected people seems to have little effect on the final outcome, as long as the initial cohort is reasonably small.

We have found that the reproductive number significantly impacts the spread of the disease but the size of the initial cohort of infected individuals has much less impact. We have also found that some values for parameters lead to unrealistic results, such as a large value for the reproductive number leads to more people contacted than the population size. Similar results occur for the logistic equation Eq. 6 since changes in the parameter *L* result in significant changes in the long-term population size, but changes in the parameter *r* have no effect on the long-term population size but can result in strange, even unrealistic results if *r* is too large. Parameters have different effects, some large and some small.

Students could also discover other effects of changes in parameters. They might explore and discover that if the reproductive number is less than 1, then the number of infected decreases, but if it is greater than 1, the number of infected will increase before decreasing.

In Table 3, the second row gives the number of newly contagious using the reproductive number 1.6. The last row lists the case rate of infection, R, for each cohort; that is, R is the average number of persons infected by each member of the cohort. R is the rational number computed by dividing the number of new cases in the next cohort by the number of new cases in the current cohort (Eggo, 2018). Just as when the reproductive number is less than 1, when R is less than 1 the contagious disease is abating.

**Table 3:** The third row is the case rate of infection, the proportion infected by current number of infected

Cohort	1	2	3	4	5	6	7	8	9
Newly infected	100	157	238	343	457	542	549	459	317
R	1.57	1.52	1.44	1.33	1.19	1.01	.84	0.69	0.59

Students should learn to look for reasons behind patterns. For example, an examination of Tables 1 and 3 along with the spreadsheet can help students see why the number of new cases may increase from cohort to cohort, but eventually decreases. In particular, there are about the same number of contagious people in cohorts 5 and 8, and therefore about the same number of contacts. But the number of susceptible persons in the population is decreasing, and so the number of vulnerable (susceptible) contacts is decreasing. After that, **as long as the parameters do not change**, both the numbers of new cases and the proportion of susceptible contacts are decreasing. This analysis shows that once the number of new cases starts decreasing, it will continue to decrease. To see that it eventually reaches zero may not be accessible to students, but students with an understanding of mathematical induction can see this by proving that any decreasing sequence of natural numbers is finite.

Finally, students could explore how the initial population size affects the spread of the disease. Students should try varying the initial population size, but keep the initial number infected at 100 and infection rate at 8(0.2) = 1.6. It would be unreasonable to compare the total number of infected for a population size of 5000 versus a population size of 1,000,000. Instead, students should compare the proportion infected, which they would discover is about the same. In other words, the initial population size seems to have little effect on the total percent eventually infected. This suggests that instead of looking at fixed population sizes, we could look at the fraction of the population contagious, susceptible and immune. Doing this would mean using decimals for the initial contagious, susceptible and immune cohorts; with the 3 decimals adding to one. Students investigating this model would be learning to compare apples to apples, that is, percent infected, not total infected, increasing their appreciation of the use of proportions.

In summary, by varying different important parameters systematically, students should gain insight into the effect each parameter has on the model. While the contagious disease model may be a little more complicated, the medicine models 3) and 7) can also be explored by changing the parameters for the rates of elimination, the rates of transmission between plasma and liver, the size of the dose, and the starting amount of medicine. Similarly, the logistic model can be explored by determining the effect resulting from the intrinsic growth rate *r* and the carrying capacity *L*. One result is that whether there is a horizontal asymptote or not depends only on the value for *r*.

Students exploring recursive models are learning how to explore as mathematicians do. In addition, students studying our contagious disease model might gain an appreciation for how mathematics is used in the fight against COVID-19, and against contagious diseases in general.

## Research questions

Previous sections discussed how we believe recursive modeling can be easily and effectively interspersed throughout the curriculum, with the potential of supporting student learning.

In summary:

- §3: a recursive model using proportions and averages in a significant context.
- §4: spreadsheets used to systematically explore a model.
- §5: connections among recursive modeling with difference equations, sequence notation, and function notation.
- §6: connections between recursion and graphical representations, particularly asymptotes.
- §7: parameters within the context of a recursive model.

While we emphasized one particular model to exemplify our ideas in some detail, in Sects. 3, 5, 6 and 7 we also noted recursive representations of linear, exponential, and other non-linear functions. These representations could be used at many places in the curriculum to support learning about these important function families. This section presents some questions, followed by brief discussions, that we hope mathematics education researchers will address. The answers to these questions might provide insight on how recursive thinking can support and broaden the school curriculum while developing students’ mathematical thinking.

o What are the different cognitive challenges for learners inherent in the recursive form compared to the closed-form?

We have given examples in which explicit solutions to recursive problems are easily developed, and in which explicit solutions are difficult or impossible to generate. What are the advantages of each? This probably depends on the models and the age of the students. For example, some practicing secondary teachers have used simple recursive problems (Bannard, 1991; Burrell et al., 1991; Reinthaler, 1997), to have their students generate closed-form linear and parabolic solutions to simple difference equations. On the other hand, for the drug models 3) and 7), finding the equilibrium and using spreadsheet graphs to explore gives most of the needed information. The closed formula for the Fibonacci sequence is both difficult to derive and does not give much information other than the limiting growth rate, while writing the sequence out by hand leads to patterns, such as the alternation between two odd numbers followed by one even number—which are easy to prove from the construction. Some equations, such as the logistic equation Eq. 6 and contagious disease model 5), generally do not have closed-form solutions so that other types of analyses must be used to gain information from the model. This leads us to ask, when a closed-form solution to a recursive model is not readily available, what are the developmental advantages of each approach to writing a recursive model, such as the Now-Next method, and sequence notation?

More generally, these considerations raise this basic question.

o How does exploring with recursive models change students’ perceptions of and attitudes toward mathematics?

Even where no closed-form solution exists, students can explore, for example noticing how asymptotes give a lot of information in the drug, contagious disease, and population models. In most math models, mathematicians cannot find closed-form solutions; so they use other methods to try to glean information from the model. Thus, students exploring such models are acting more like mathematicians. While some students may enjoy manipulating formulas, other students may become engaged with mathematics through exploring meaningful contexts and reflecting on the structure of the results of a model, especially when technology such as spreadsheets automates tedious calculations.

o How are students’ algebraic and graphic skills enhanced by learning to move among representations in recursive models? Does analyzing recursive models encourage students to be more flexible and move fluidly among different modes of analysis?

Related to the previous question, there is a close relationship between recursive models, algebra, and graphs. As discussed in §2, there is evidence that recursive thinking supports younger students’ algebraic understanding, such as observing a relationship between repeated addition and linear functions. In what other ways does a recursive view strengthen students’ understanding of linear functions? Given this evidence related to linear functions, it seems surprising that there has been little work looking at how a recursive view relates to students’ understanding of exponential functions.

Even more, recursive models involve exploring relationships among formulas, parameters, asymptotes, and equilibria, within contextual situations. In what ways do such recursive explorations further enhance students’ algebraic and graphical understanding? Does considering graphs within a context lead to a better understanding of the relationships among the different variables and parameters in a problem? For example, determining the effects of a parameter on an expression is often difficult, as in determining that for the quadratic function

$$y = \left( {x + a} \right)^{2} + b$$

it may be unintuitive to discover that increases in the parameter $b$ results in the parabola being shifted upward while increases in $a$ results in the parabola shifting to the left. On the other hand, in recursive models, there is a contextual relationship between the parameter and the graph that makes the results more intuitive. For example, increasing the infection rate increases the horizontal asymptote for the total infected while increasing the elimination rate for a drug results in the horizontal asymptote decreasing.

The fact that recursion involves moving among context, formulas, and graphs may increase students’ flexibility in problem solving. We note that Alcock and Weber (2010) found that college students tend to lack flexibility in their approaches to proving, preferring either a referential or syntactic approach. Will working recursively increase students’ mathematical flexibility?

o How does the contextual use of simple probabilities and proportions in the development of models affect students’ understandings of proportional reasoning, probability, and statistics?

In what ways will recursive modeling support students’ understanding of statistics? The model of the spread of a contagious disease predicts exact values similar to predicting that half of some number of flips of a coin will be heads. On the other hand, a statistical result predicts a result with some degree of variation. These are two different ways to explore the same situation. How will students react to these different approaches?

In addition to comparing deterministic versus statistical results, recursive models often use proportional reasoning, such as in our model of contagious disease spread. Not only is proportional reasoning used in development of models like this, but the results may include predictions of averages and expected values.

## Discussion

We believe the use of difference equations and spreadsheets to explore and analyze naturally recursive real-world situations has the potential to enhance students’ understanding of and attitudes toward mathematical reasoning and applications.

While our primary model is the study of contagious diseases, we identified other topics that could be used. We focused on contagious diseases to show one way combining recursion and current affairs can engage students in mathematics.

Various sources of recursive problems are appropriate for study by students at lower levels of school (DeBellis et al., 2009; Malkevitch & Meyer, 1974; Seymour & Shedd, 1973). The curriculum project Core Plus incorporates recursive modeling throughout its texts (Hart, 1997). Throughout this article, we mentioned other sources of problems appropriate at the middle and secondary levels (Cornell & Siegried, 1991; Hart et al., 2008; Sandefur, 1997; Sandefur et al., 2018). It is a little more difficult to find truly applied models similar to our disease model. One source of applications of difference equations with examples similar to our model is Sandefur (2002). Its examples include the buildup and elimination of caffeine or alcohol, the effects of selection and mutation on the genetic makeup of a population, and sustainable harvesting models. They all can be adapted to the middle or secondary mathematics classroom, similar to the ways we adapted the contagious disease model in this paper.

We argued that students’ early exposure to recursive patterns, such as sequences or repeated patterns of colors or shapes, should be built upon—not dropped as it currently is. To do this effectively, studies should be conducted to determine how the different forms of recursion can develop sound habits of thinking and reasoning. What models and approaches are most age appropriate? How should students progress from verbal descriptions (add 4) to the Now-Next approach to sequence notation and to difference equations? At what point and how should spreadsheets be introduced? How do recursive models support students’ understanding of basic arithmetic operations, and of probability and proportions? Do recursive models support students’ understanding of and ability to interpret graphs?

The questions just suggested are basic. We believe there is even more power from introducing realistic applied contexts. The idea behind our disease model was to show that, with some basic mathematics (addition, proportions, multiplication) and spreadsheets, students at a variety of levels can explore meaningful and significant examples, helping to develop student agency. It not only allows them to see how the mathematics they are learning is used in practice, it lets them experience mathematics by developing reasonable assumptions and the corresponding mathematical relationships. That is, students can begin to have a sense of the power of mathematics to help them see meaningful patterns in real-world situations.

Realistic recursive models provide a rich area for educational studies on the effects of these models on students’ learning and valuing mathematics. We believe these applied problems are likely to have a positive impact on students’ attitudes toward mathematics by letting them experience real mathematics. How does studying these problems improve students’ abilities to reason and explore? How does having students write clearly about the justifications for and the shortcomings of their assumptions affect their reasoning and mathematical communication skills? How does explaining their conclusions based on their explorations improve their writing and reasoning?

In short, we believe significant benefits can result from the increased introduction of recursion in the mathematic curriculum. We hope that the mathematical education community studies these possibilities in the near future.
